# Novel Approach to Phase-Sensitive Optical Time-Domain Reflectometry Response Analysis with Machine Learning Methods

**DOI:** 10.3390/s24051656

**Published:** 2024-03-04

**Authors:** Vasily A. Yatseev, Oleg V. Butov, Alexey B. Pnev

**Affiliations:** 1 Kotelnikov Institute of Radioengineering and Electronics of Russian Academy of Science, 125009 Moscow, Russia; obutov@mail.ru; 2 Scientific Educational Centre “Photonics and IR Engineering”, Bauman Moscow State Technical University, 105005 Moscow, Russia; apniov@gmail.com

**Keywords:** distributed fiber optic sensing, chirped-OTDR, phase demodulation, machine learning

## Abstract

This paper is dedicated to the investigation of the metrological properties of phase-sensitive reflectometric measurement systems, with a particular focus on addressing the non-uniformity of responses along optical fibers. The authors highlight challenges associated with the stochastic distribution of Rayleigh reflectors in fiber optic systems and propose a methodology for assessing response non-uniformity using both cross-correlation algorithms and machine learning approaches, using chirped-reflectometry as an example. The experimental process involves simulating deformation impact by altering the light source’s wavelength and utilizing a chirped-reflectometer to estimate response non-uniformity. This paper also includes a comparison of results obtained from cross-correlation and neural network-based algorithms, revealing that the latter offers more than 34% improvement in accuracy when measuring phase differences. In conclusion, the study demonstrates how this methodology effectively evaluates response non-uniformity along different sections of optical fibers.

## 1. Introduction

Since the 1970s, the rapid development of fiber optics has led to significant breakthroughs in telecommunication technologies. In a short period of time, the use of fiber optics extended beyond the realm of communications. Due to a range of unique properties, fiber optic sensing is actively being implemented. Fiber optic sensors have emerged, including distributed measurement systems operating on reflectometry principles [1,2]. Unlike traditional point sensors, these systems can collect measurement data from tens of kilometers of optical fiber at a high frequency and accurately determine the point of impact. Such systems have found applications in areas such as pipeline protection [3], oil well monitoring [4], railway transport movement control [5], aviation [6], and perimeter security systems [7]. Typically, these systems measure physical properties such as temperature, mechanical deformation, and vibro-acoustic signals. However, they can also be adapted to detect the presence of chemical substances [8] and much more.

As the technology evolved, the focus shifted from simple monitoring systems, which only qualitatively inform about the point of impact on the fiber, to full-fledged measurement systems providing high metrological characteristics. In this regard, the task of researching and improving metrological properties remains relevant. It is also essential to consider that fiber optic distributed measurement systems differ from point systems with their specific features, one of which is the heterogeneity of characteristics along the fiber, associated with the stochastic distribution of reflectors.

To build such measurement systems, it is necessary to conduct research aimed at understanding and taking into account the heterogeneity of characteristics along the fiber, as well as developing methods and algorithms that allow for compensating and minimizing the influence of the stochastic distribution of Rayleigh reflectors. Such an approach will enhance the accuracy and reliability of measurements in fiber optic distributed measurement systems, opening new opportunities for their application in areas requiring in-phase detection from different sections of the fiber, for example, seismic signal processing, the detection of extended acoustic waves, and more.

### 1.1. Rayleigh Reflectometry

There are several types of reflectometers based on different types of backscattered light signals, but the most common is the phase-sensitive reflectometer based on the interference of signals from Rayleigh reflectors in the fiber. There are two basic approaches to building reflectometric systems: OTDR (Optical Time-Domain Reflectometry) and OFDR (Optical Frequency-Domain Reflectometry).

#### 1.1.1. OTDR

A series of high-intensity light pulses are sent into the fiber and the scattered light is detected in the reverse direction. The analysis is based on measuring the time delay and intensity of the backscattered light. The spatial resolution of such a reflectometer typically ranges from a few meters, depending on the characteristics of the pulses. OTDR has a large dynamic range, making it suitable for testing long stretches of optical fiber [9]. An improved version of this reflectometer is the phase-sensitive reflectometer ϕ-OTDR [10], which uses a coherent light source. It detects the distribution of the total interference pattern from reflectors during the passage of a light pulse. This reflectometer has high sensitivity due to interference effects. Sometimes, to enhance the quality of the interference signal, artificial reflectors [11] or Bragg gratings [12] are embedded in the fiber.

#### 1.1.2. OFDR

A continuous-wave laser source with a tunable frequency is used. The backscattered light is collected from the fiber and mixed with the reference signal of the local oscillator. Signal analysis is based on information about the beat frequencies of the interference signal by applying a Fourier transform. OFDR has high spatial resolution, usually from micrometers to millimeters at measurement lengths of about a hundreds meters; it has a higher signal-to-noise ratio, making it more suitable for high-resolution applications and short distances. For long distances, the characteristics of OFDR degrade sharply [13].

### 1.2. Chirped-Reflectometer

There are also approaches that combine OTDR and OFDR methods, which merge the advantages of both methods, one of which is chirped pulse time-domain reflectometry (CP-OTDR) [14]. CP-OTDR is a variant of the standard coherent OTDR reflectometer, in which an optical pulse with a varying frequency of radiation is used. Using a chirped pulse offers several advantages compared to conventional reflectometers that use single-frequency pulses: improved resolution, reduced “dead” zone sizes, extended distance range, and a higher signal-to-noise ratio. Moreover, this type of reflectometer belongs to direct detection reflectometers, i.e., without the use of a local oscillator, making them technically simple, unlike homodyne and heterodyne optical schemes. At the same time, such reflectometers, thanks to the correlational signal processing, are capable of performing phase measurements using a single receiver.

### 1.3. Measurement of Response Non-Uniformity along the Fiber

As mentioned above, for distributed fiber optic measurement systems, unlike point sensors, there are specific aspects such as non-uniformity of sensitivity along the fiber, cross-talk between adjacent measuring channels, response blurring from point impact, etc., that affect the metrological parameters: measurement range, sensitivity threshold, and accuracy. Despite its importance, the literature does not sufficiently address the metrological parameters of distributed fiber measurement systems. Usually, it is only mentioned that there are problems with response homogeneity and the presence of interference fading [15]. Many studies describe setups designed to investigate the accuracy characteristics of reflectometers at one or several points along the fiber, for example, using piezoactuators with fiber wound around them [16]. However, with this approach, studying the homogeneity of the response along the fiber is complicated by the need to create identical conditions of impact on the fiber at multiple points. It is practically impossible to have several hundred coils with absolutely identical winding conditions. The influence of adhesives, different adhesion forces with the piezoactuator, the need to control tension, and other factors can significantly affect measurements, even if the piezoactuators themselves have identical characteristics. When using temperature effects on the fiber, there are also technical difficulties associated with ensuring the uniformity of the temperature field along the fiber length, especially at large measurement lengths [17]. A common drawback is the difficulty in distinguishing between temperature and deformation effects. This complicates the accurate determination of which specific influence (temperature or deformation) causes changes in the characteristics of the reflected signal, and which changes are the result of external noise. Therefore, to study the response along a long optical fiber line, it is not enough to use standard methods to evaluate the system’s reaction using deformation or temperature effects, as the non-uniformity of the response along the fiber when using a Rayleigh phase-sensitive reflectometer is due to the random distribution of Rayleigh reflectors along the fiber.

### 1.4. Reception Schemes and Signal Processing from the Phase-Sensitive Reflectometer

For the phase-sensitive reflectometer, various algorithms and optical schemes are used to demodulate the phase of the interference signal. These could be schemes with a single photoreceiver, as in the case of the chirped-reflectometer [14], or with two receivers (IQ-hybrid) [18], or three receivers (3 × 3 coupler) and other demodulation schemes [10].

To work with a complex signal and find hidden correlations, neural algorithms, which had already become widespread [19], have recently been more widely used. Similar algorithms are also used for processing data obtained from fiber optic sensors [20] to process signals in the OTDR reflectometer [21,22]. In the phase-sensitive Rayleigh reflectometer, neural algorithms are used to reduce noise [23] and, especially widely, for the classification of vibration events [24]. We believe that these artificial neuron-based algorithms can be promising for detecting complex signals, in particular, for calculating phase differences based on reflectograms.

This work is dedicated to the study of the methodology for estimating the metrological properties of phase-sensitive reflectometric measurement systems, with an emphasis on the quantitative study of response homogeneity along the optical fiber. In particular, based on this methodology, comparisons of reflectogram processing methods are made depending on signal processing algorithms—traditional algorithms and machine learning-based algorithms.

Our work presents a method for simulating impacts of deformation and temperature, which allows for further metrological analysis and the collection of large data sets for training neural networks. To our knowledge, there are no other studies that have presented a similar methodology for phase-sensitive reflectometric systems. The effectiveness of our method is demonstrated by the formulas used, as well as by references to works on cross-correlation methods [25,26] used for measuring deformation and temperature. Additionally, there are no studies on phase demodulation in reflectometric systems using natural Rayleigh reflectors with neural algorithms. Collecting data for training neural algorithms from different fiber sections is a non-trivial task, and our method is specifically designed to obtain such data.

## 2. Materials and Methods

Interference within a light pulse propagating through the fiber is described by the cumulative pairwise interference pattern from the reflectors, calculated using Equation (1):

(1) $$I\left(t\right)=I_{0}\sum_{m=1}^{M}r_{m}^{2}+2I_{0}\sum_{i=1}^{M-1}\sum_{j=i+1}^{M}r_{i}r_{j}\cos\left(\phi_{i}-\phi_{j}\right),$$

where I(t) is the intensity value for the reflectogram, $I_{0}$ is the intensity of the incident radiation, $r_{i}$ is the reflection coefficient of the *i*-th scatterer, *M* is the number of reflectors falling within the light pulse, and $\phi_{i}-\phi_{j}$ is the phase difference of light waves for the *i*-th and *j*-th reflectors. In turn, the phase difference is expressed as:

(2) $$\phi_{i}-\phi_{j}=4\pi n\left(\frac{L_{ij}}{\lambda }\right),$$

where *n* is the refractive index, $L_{ij}$ is the distance between the *i*-th and *j*-th reflectors, and λ is the wavelength.

When external influences such as deformation or temperature occur, the optical paths $nL_{ij}$ change. Since phase is a dimensionless quantity, the change in the optical path is normalized to the wavelength of the radiation, thus allowing the change in the optical path to be compensated by altering the wavelength and vice versa. From these considerations, the well-known relation used for Bragg gratings in SMF28 fiber, Δλ/λ=0.78μϵ, is derived [27,28]. This ratio determines the relative shift of the Bragg peak reflection Δλ/λ under deformation ϵ. The same equation applies not only in the presence of periodic structures in the fiber, but also in a random grating that can be formed by Rayleigh reflectors, where the whole reflection spectrum shifts [29].

The method of restoring the original appearance of the reflectogram in response to temperature or deformation by adjusting the wavelength is the basis of the cross-correlation method in the frequency [26] and spatial domains [30].

For example, when stretching the fiber by 1 μm, a similar effect on the reflectogram can be achieved by shifting the 1.5 μm wavelength by 1.5 pm, and vice versa. This phenomenon is used in this work to study the response of the reflectometric system, simulating external influence by changing the wavelength of the laser.

**Figure 1:**
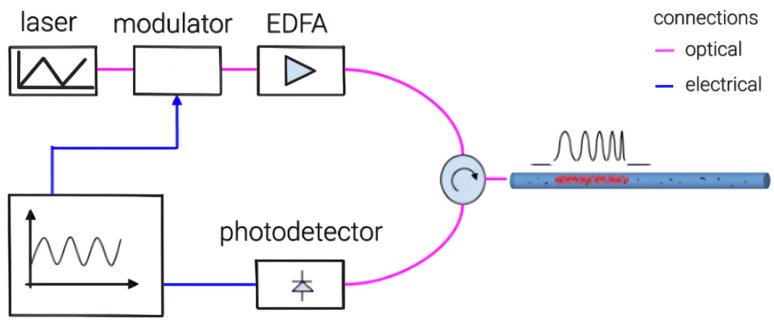
Optical scheme of the scanning reflectometer.

The optical scheme of the scanning reflectometer used in our experiments is shown in Figure 1.

The scanning chirped-reflectometer setup includes the following parameters.(1) NKT X15 erbium laser (made by NKT Photonics A/S): linewidth up to 1 kHz; wavelength of 1550 nm; wavelength fast linear modulation range of 500 MHz (1.6 pm); low phase noise [31]; the Allan deviation for the X15 laser module stays below $10^{-8}$. (2) SOA (semiconductor optical amplifier, IPSAD1531, Inphenix Inc., Livermore, CA, USA)-based modulator: pulse duration of 70 ns; repetition rate of 2 kHz. (3) EDFA (erbium-doped fiber amplifier) booster: output power up to 200 mW in the pulse, with a filter for suppressing noise from the amplified spontaneous emission. (4) SMF28-type measuring fiber. (5) PIN photodetector with analog-to-digital converter (ADC): sampling frequency of 100 MHz, allowing for the reading of every meter. Thus, a 70 ns pulse corresponds to 7 m of the received reflectogram and 7 values recorded by the ADC, which determines the distance resolution. The scheme operates as follows: the laser emits high-coherence continuous radiation, which is modulated by an optical amplifier (SOA), converting it into a series of chirped pulses. These pulses are characterized by a change in frequency over time and propagate through the fiber optic cable, reflecting off inhomogeneities in the fiber and receiving signals by the PIN photodiod, which converts the received optical signal into an electrical one, which is then digitized by the ADC.

The setup essentially consists of an OTDR, where a laser capable of tuning the wavelength up to 1.35 pm from the central wavelength was used as the light source. This allows for simulating the effects of deformation or temperature on the fiber. In our case, a chirped-reflectometer was used; however, it could be any other type of OTDR reflectometer that allows for phase recovery.

Frequency scanning was performed according to a saw-tooth law with frequencies ranging from 0.1 to 10 Hz. This frequency is significantly lower than the frequency of the probing pulses of the kilohertz range, which means that a reflectogram corresponds to the one laser wavelength. At the same time, each subsequent reflectogram corresponds to different wavelengths of the radiation source.

When an impact, such as deformation or temperature change, is applied to the fiber, a shift in a part of the reflectogram occurs, allowing for the determination of the location and intensity of the impact.

**Figure 2:**
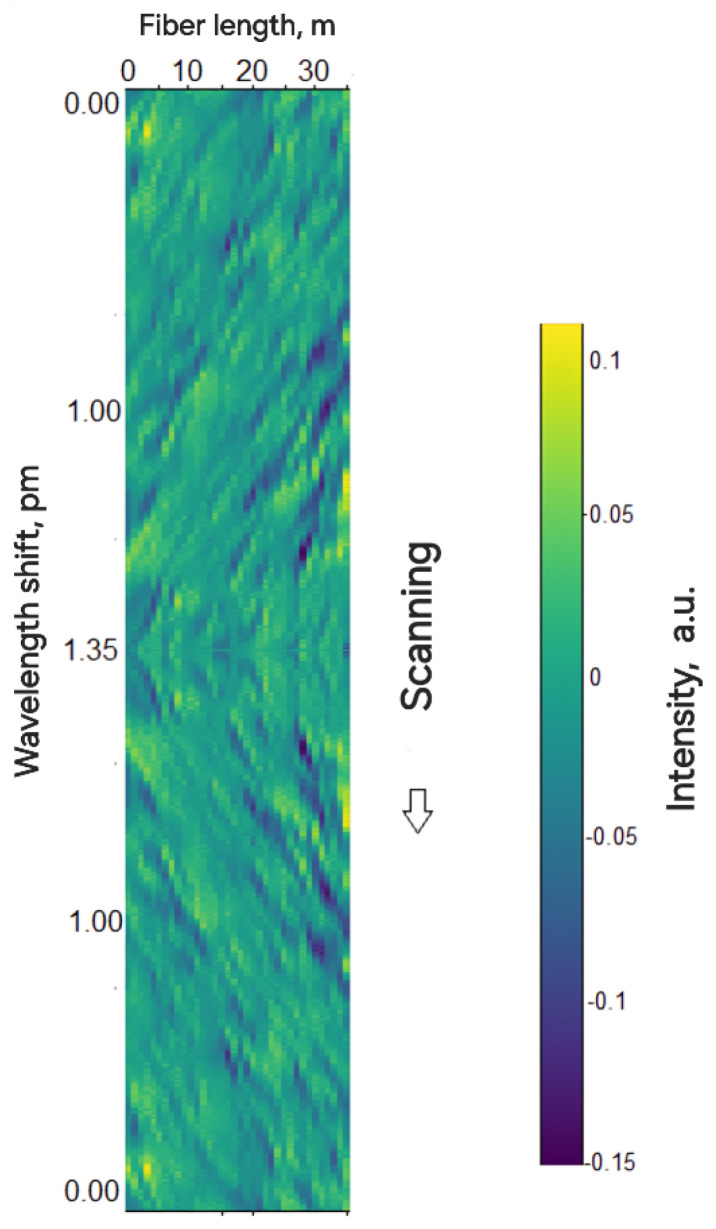
“Waterfall” representation of reflectograms.

Figure 2 displays a series of reflectograms stacked together, obtained during the saw-tooth wavelength sweeping process for a 35 m fiber section.

**Figure 3:**
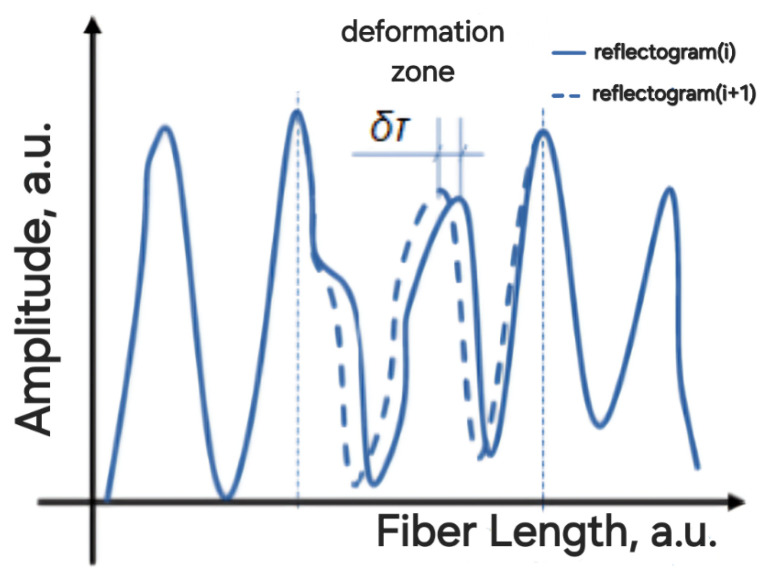
Change in the reflectogram detected by the chirped-reflectometer when deformation applied on the fiber.

The diagram includes series of 1000 reflectograms, with a wavelength shift of 2.7 fm between each other. The first 500 reflectograms were taken with a positive wavelength shift, reaching 1.35 pm from the initial wavelength of 1550 nm, after which a reverse shift occurred. Figure 2 shows a displacement of the interference fringes, characteristic of a chirped-reflectometer, both to the left in the first half and right in the last half. This drift of the interference fringes, although having an irregular structure due to the stochastic distribution of reflectors in the fiber, is an important indicator of changes in the optical path, caused by external impacts on the fiber or changes in the wavelength. To assess the impact on the fiber using the chirped-reflectometer, it is necessary to measure the shift in the reflectogram for the corresponding section (see Figure 3).

The shift value of the reflectogram, δτ, corresponding to the area of mechanical impact on the fiber, allows for the determination of the sign and magnitude of the deformation. In our case, when we simulate the impact on the fiber by changing the wavelength of the radiation source, the reflectogram shifts along the entire length of the fiber (Figure 2).

### 2.1. Cross-Correlation Algorithm

For processing the signal from the phase-sensitive reflectometer, the cross-correlation (CC) algorithm is commonly employed. This method detects the shift of interference fringes between two consecutive reflectograms, $I_{1}$ and $I_{2}$ (see Equation (3)), by finding the correlation magnitude between the reflectograms taken from the same place at consecutive moments in time with a correlation window $\tau_{corr}$, usually equal to the pulse duration [25]:

(3) $$\Lambda \left(t\right)=\max\left(\mathrm{correlation}\left[I_{1}\left(t-\frac{\tau_{\mathrm{corr}}}{2},t+\frac{\tau_{\mathrm{corr}}}{2}\right),I_{2}\left(t-\frac{\tau_{\mathrm{corr}}}{2},t+\frac{\tau_{\mathrm{corr}}}{2}\right)\right]\right),$$

The shift in the correlation peak relative to the two consecutive reflectograms indicates the magnitude and direction of the deformation or temperature. Despite the relative simplicity of the method, it has several drawbacks, one of which is the presence of outliers that need to be filtered out. In typical cases, 10% of phase measurements are incorrect and require adjustments [32]. This method establishes a baseline level of the algorithm quality, against which the neural network-based algorithms were compared.

### 2.2. Machine Learning Method

Neural networks [33] are capable of processing large volumes of data, identifying complex patterns, and adapting to changing conditions, making them ideal for analyzing reflectograms and detecting subtle phase changes caused by external influences. The application of neural networks begins with training the model on a large dataset, including reflectograms with known characteristics of impacts. For data collection necessary for training the machine learning algorithm, we used the same experimental setup as in Figure 1. The main advantage of this setup is that it not only provides a large volume of data, but also allows for readings from different sections of the fiber. These data can be mixed together, which helps prevent the algorithm from overfitting to specific features of a particular section of the fiber.

The training dataset was formed as follows. From two consecutive reflectograms with a wavelength shift of 0.27 pm between them, segments of seven values each were selected, corresponding to the correlation window used in the cross-correlation method and equal to the pulse length in the fiber. Then, the 7 × 7 matrix, composed of pairwise multiplied values of these reflectogram segments, was fed into the neural network. These data were then processed by two dense layers, each containing 9 neurons. In each of these layers, a sigmoid activation function was used, while the output neuron functioned with linear activation [19].

**Figure 4:**
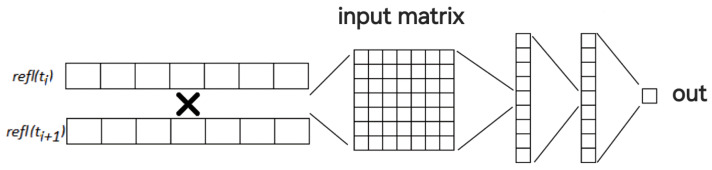
Phase decomposition neural network topology.

After several iterations and improvements, the following neural network scheme was proposed (Figure 4).

This approach allowed for the expansion of the cross-correlation function by including additional multipliers, which contributed to improved accuracy. Although the primary objective of this work was not to achieve the most optimal neural network configuration, during the optimization process, various neural network configurations were explored, involving different numbers of neurons and a range of activation functions. The selected scheme near to the optimal was not overly complex or demanding in terms of training time, yet it demonstrated acceptable results, comparable to the best options in this neural network topology. During the training process, the output of the neural network was also fed with a value corresponding to the wavelength shift. For training, 4000 values collected from different sections of the fiber were used, which significantly exceeded the necessary number of parameters for training. No signs of overfitting were detected.

## 3. **Results and Discussion**

**Figure 5:**
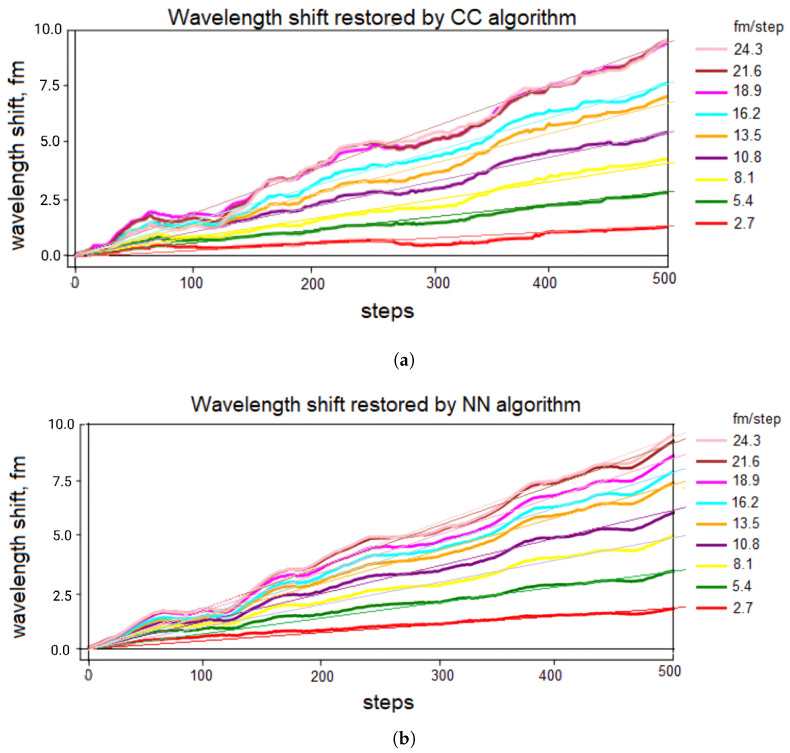
System responseto a linearly increasing signal obtained using algorithms: (**a**) cross-correlation (CC), (**b**) based on a neural network (NN), where 2.7…24.3 [fm/step]—the rate of increase in the radiation wavelength.

The results obtained using these algorithms demonstrate a close-to-linear response to the linear changes in the radiation wavelengths at different rates, from 2.7 fm/step to 24.3 fm/step, as shown in Figure 5. Each step represents the number of the reflectogram recorded with the corresponding wavelength shift.

It can be seen that the algorithm based on the neural network provides a more linearly approximated reconstructed signal compared to the cross-correlation-based algorithm. Moreover, for both algorithms, it is true that at low rates of influence, the signal shape is closer to linear, and at high rates, it becomes more nonlinear. Also, at high speeds, a decrease in sensitivity with some saturation effect is noticeable, which defines the dynamic range limits for the rate of influence at 15 fm/step corresponding to 1 nϵ/step. For our optical scheme, with a 2 kHz pulse repetition rate, the maximum linear signal slope increase achievable by these algorithms is 1 nϵ per 0.5 ms, which equates to 2 μϵ per second. For these demodulation schemes, it is crucial to operate within this range to decompose the phase linearly.

### Evaluation of the Non-Uniformity of the Response along the Fiber

**Figure 6:**
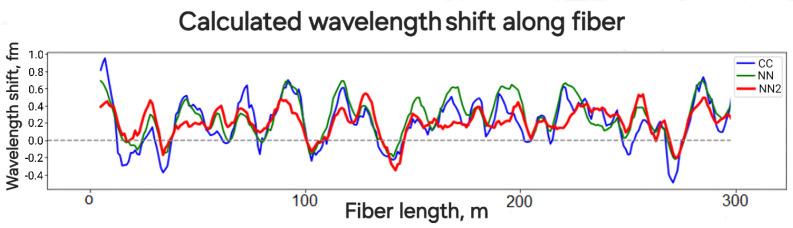
System response along the cable to a change in wavelength by 1 fm calculated by NN1, NN2, and CC algorithms.

For a quantitative assessment of non-uniformity, we analyzed the reflectogram using cross-correlation and machine learning algorithms to determine the response to a change in wavelength by 1 fm. After obtaining the response along the fiber, we applied a moving average with a window of seven values, corresponding to the pulse length. In addition to the neural network-based algorithm (NN1) that trained on two consecutive reflectograms, for comparison, we added data obtained with a neural algorithm (NN2) that takes into account not two, but three consecutive reflectograms. This increases the measurement accuracy since it considers more data. The data are shown in Figure 6.

Although the curves behave similarly, the algorithms based on neural analysis (NN1, NN2) demonstrate better results compared to the cross-correlation algorithm (CC). In particular, the NN1 and NN2 curves cross the zero line less frequently than the CC curve, indicating that the signal is less susceptible to interference fading and reverse sensitivity, which show a negative signal when influenced positively and vice versa. In quantitative terms, the root-mean-squared deviation (RMSD) processed using the CC, NN1, NN2 algorithms are 0.39 fm, 0.26 fm, and 0.22 fm, respectively, with a change in wavelength by 1 fm. Thus, the proposed methodology allows for the assessment of response non-uniformity and demonstrates that the application of machine learning algorithms significantly improves the accuracy of phase measurement in conditions of stochastic distribution of reflectors in the fiber.

## 4. Conclusions

This work presents a method for simulating the effect of deformation on fiber by changing the wavelength. This approach provides higher accuracy compared to direct deformation impact, as it is not influenced by transducers, adhesives, fixings, and temperature, with accuracy being determined solely by the high precision of interferometric wavelength measurement. The method also allows for data collection from different sections of the fiber and creating large volumes of data, which is important for testing and comparing various phase demodulation algorithms. Particularly significant is the application of this setup for collecting data for algorithms based on neural network. The results highlighted substantial accuracy improvements achieved by neural network algorithms, with standard deviations of 0.26 fm for two-reflectogram neural networks and 0.22 fm for three-reflectogram neural networks, in contrast to the 0.39 fm observed with the cross-correlation method. Furthermore, the study assessed the deformation rate range conducive to distortion-free signal recovery in chirped-reflectometry, revealing a range of 0 to 2 microstrain/s.

## Data Availability

Data accessible upon request.

## Abbreviations

The following abbreviations are used in this manuscript:

OTDR	Optical Time-Domain Reflectometry
OFDR	Optical Frequency-Domain Reflectometry
CP-OTDR	Chirped Pulse Time-Domain Reflectometry
EDFA	Erbium-Doped Fiber Amplifier
SOA	Semiconductor Optical Amplifier
RMSD	Root-Mean-Squared Deviation
CC	Cross-Correlation
NN	Neural Network
NN1	Neural Network of 7 × 7 Input Matrix
NN2	Neural Network of 3 × 7 × 7 Input Matrix
